# Double-pulsed wave packets in spontaneous radiation from a tandem undulator

**DOI:** 10.1038/s41598-022-13684-2

**Published:** 2022-06-11

**Authors:** T. Kaneyasu, M. Hosaka, A. Mano, Y. Takashima, M. Fujimoto, E. Salehi, H. Iwayama, Y. Hikosaka, M. Katoh

**Affiliations:** 1 SAGA Light Source, Tosu, 841-0005 Japan; 2 Synchrotron Radiation Research Center, Nagoya University, Nagoya, 464-8603 Japan; 3 Institute for Molecular Science, Okazaki, 444-8585 Japan; 4 Sokendai (The Graduate University for Advanced Studies), Okazaki, 444-8585 Japan; 5 Institute of Liberal Arts and Sciences, University of Toyama, Toyama, 930-0194 Japan; 6 Hiroshima Synchrotron Radiation Center, Hiroshima University, Higashi-Hiroshima, 739-0046 Japan; 7 Present Address: National Synchrotron Radiation Laboratory, University of Science and Technology of China, Hefei, 230029 China

**Keywords:** Atomic and molecular interactions with photons, X-rays, Free-electron lasers, Ultrafast lasers

## Abstract

We verify that each wave packet of spontaneous radiation from two undulators placed in series has a double-pulsed temporal profile with pulse spacing which can be controlled at the attosecond level. Using a Mach–Zehnder interferometer operating at ultraviolet wavelengths, we obtain the autocorrelation trace for the spontaneous radiation from the tandem undulator. The results clearly show that the wave packet has a double-pulsed structure, consisting of a pair of 10-cycle oscillations with a variable separation. We also report the characterization of the time delay between the double-pulsed components in different wavelength regimes. The excellent agreement between the independent measurements confirms that a tandem undulator can be used to produce double-pulsed wave packets at arbitrary wavelength.

## Introduction

Electromagnetic radiation from ultra-relativistic electrons plays many important roles in modern science and technology. In particular, synchrotron radiation provides indispensable probes for a wide range of research fields. In modern synchrotron light sources, a device called an undulator is widely used, which subjects the orbiting electrons to a periodically alternating magnetic field. In this device, the ultra-relativistic electrons execute an undulating motion and radiate quasi-monochromatic light^1^. The undulator radiation may be understood as follows; in the reference frame moving with the average velocity of the electrons, the electrons execute a harmonic oscillation and radiate monochromatic radiation. In the laboratory frame, the wavelength is compressed by the square of Lorentz factor due to the relativistic Doppler effect. The typical Lorentz factors for the electrons in synchrotron light sources are around 10^3^–10^4^. Therefore, the period of the undulating motion of the electrons of the order of centimeters is converted to radiation wavelengths of nanometers or Angstroms. This implies that, by controlling the electron motion in the centimeter scale, one can control the properties of the radiation waveform in the nanometer or Angstrom scale. However, until recently, this possibility has rarely been considered, because synchrotron radiation consists of the superposition of spontaneous radiation from large numbers of electrons, and the individual characteristic waveforms are buried in the incoherent mixture of the wave packets.

A tandem undulator, which consists of two undulators placed in series, is a powerful light source, bringing unique abilities—for example, polarization switching^2–4^ and combination of different wavelength regimes^5,6^– in spectroscopic studies of matter. Moreover, interference between the radiation from the two undulators can be controlled by a phase shifter magnet which delays the electron motion, and is used to control the polarization of light in the crossed undulator scheme^7–10^ and to verify the generation of structured light such as vortex beams^11–13^ and vector beams^14^ in synchrotron light sources. The waveform of each wave packet constituting the radiation has a double-pulsed time structure, reflecting the sequential undulating motion of electrons traveling through the tandem undulator. The production of these double-pulsed wave packets has been indirectly demonstrated using light-matter interaction, where quantum interferences between the electron wave packets launched in atoms have been demonstrated^15–18^. Up to the present, however, the double-pulsed structures of individual wave packets has not directly been verified by any optical method, presumably because it has been thought that any optical verification is impossible due to the incoherent nature of the spontaneous radiation.

In this paper, we report on a fully-optical characterization of double-pulsed wave packets emitted by individual relativistic electrons passing through a tandem undulator in a synchrotron light source. We report on the first order autocorrelation measurement by using a Mach–Zehnder (MZ) interferometer in the ultraviolet (UV) wavelength regime. The autocorrelation function directly reflects the waveform of the double-pulsed wave packet defined by the periodic magnetic field on the electron orbit formed by the undulator. The number of magnetic periods in the undulator determines the cycle number of the wave packet and a phase-shifter magnet acts as a tuning knob for the attosecond control of the time separation (delay) between the double-pulsed components. In addition, we measured a frequency-domain spectrum which shows an interference pattern depending on the time delay. This can be used as alternative approach for probing the temporal structure of the wave packet. Further, to confirm the ability of the tandem undulator to control the radiation waveform at arbitrary wavelengths, we characterized the double-pulsed wave packet in the extreme ultraviolet (XUV) regime. Since the autocorrelation measurement becomes difficult at shorter wavelengths, we only measured the time delay by observing the quantum interference in an atomic system resulting from the sequential interaction of atom with the double-pulsed components of the wave packet.

## Results and discussion

### Autocorrelation for spontaneous radiation

**Figure 1:**
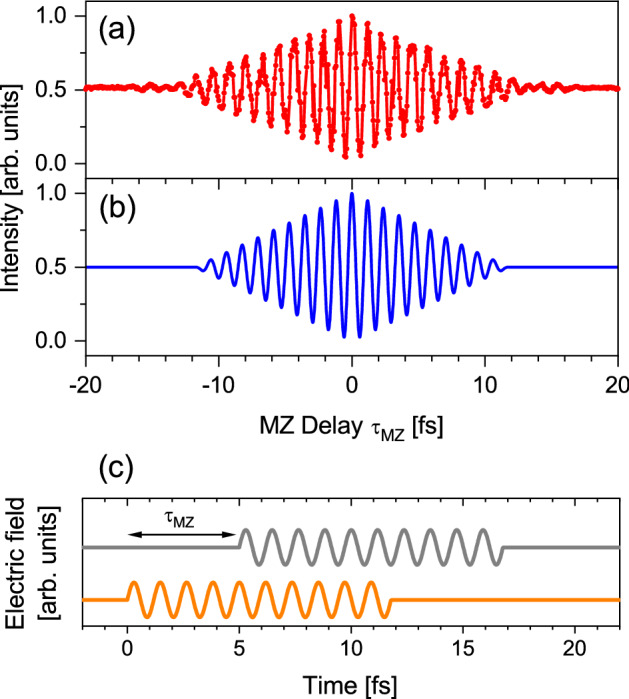
Autocorrelation measurement of spontaneous radiation from the downstream undulator. The wavelength of the fundamental undulator radiation was set to 357 nm. (**a**) Measurement. (**b**) Calculated autocorrelation function assuming a 10-cycle sinusoidal wave at 357 nm wavelength. (**c**) Waveform of the wave packet (orange) and its delayed replica (gray) used in the calculation. As an example, the waveform for MZ delay τ_MZ_ = 5 fs is shown.

Prior to the spontaneous radiation from the tandem undulator, we first consider the spontaneous radiation from a single undulator. Figure 1a shows the interferogram measured for the radiation from the downstream undulator only, using the MZ interferometer (see “Methods”). The central wavelength of the undulator radiation was set to 357 nm. The interferogram exhibits oscillations of about 20 cycles with a temporal period of around 1.2 fs, with a triangular-shaped envelope. The appearance of the oscillating feature in the interferogram is a manifestation of the temporal coherence in the spontaneous radiation from numerous electrons. The observed feature can be understood as the autocorrelation trace of the waveform of the wave packet emitted by a single relativistic electron. This is a consequence of statistically averaged measurement for numerous wave packets in which cross terms between the wave packets associated with different electrons are cancelled out^19,20^.

The autocorrelation function for an *N*-cycle sinusoidal wave packet is given by

1 $$\begin{aligned} & F\left( {\tau_{MZ} } \right) \propto \mathop \smallint \limits_{ - \infty }^{ + \infty } \left| {E\left( t \right) + E\left( {t - \tau_{MZ} } \right)} \right|^{2} dt \\ & \quad = \left\{ {\begin{array}{*{20}c} {NT} & {NT \le \tau_{MZ} } \\ {NT + \left( {NT - \tau_{MZ} } \right)\cos \omega \tau_{MZ} + \frac{1}{\omega }\sin \omega \tau_{MZ} } & {0 \le \tau_{MZ} < NT} \\ {NT + \left( {NT + \tau_{MZ} } \right)\cos \omega \tau_{MZ} - \frac{1}{\omega }\sin \omega \tau_{MZ} } & { - NT \le \tau_{MZ} < 0} \\ {NT} & {\tau_{MZ} < - NT} \\ \end{array} } \right. \\ \end{aligned}$$

where *E*(*t*) is the electric field of the wave packet expressed by sin*ωt* (0 ≤ *t* < *NT*), and *T* and ω are the temporal period and angular frequency of the radiation field, respectively. This autocorrelation function consists of 2*N*-cylce oscillations with a triangular envelope. Figure 1b presents the calculation results using Eq. (1), where the interfering wave packet is assumed to have the form of 10-cycle sinusoidal wave at 357 nm wavelength (depicted in Fig. 1c). It should be noted that, in the present experimental conditions, the undulator radiation actually also contains high harmonics, meaning that the waveform of wave packet should be described by a 10-cycle spikey field^1^. However, it is reasonable to assume a sinusoidal wave packet to calculate the autocorrelation trace obtained by the MZ interferometer because we only observed the fundamental radiation. The calculation well reproduces the experimentally-observed features in the interferogram. The observed autocorrelation trace can be thus allocated to the interference of the wave packet whose waveform is characterized by the 10-cycle magnetic field formed by the undulator. We see slight deviations between the measurement and the calculation at large delays. This can be attributed to the contribution of nonsinusoidal periodic magnetic field in the undulator formed by the end structures of the undulator magnets.

The present autocorrelation measurement does not allow for phase reconstruction. However, in this case, the waveform of the electromagnetic radiation should be simply determined by the undulating motion of the electron. The measured autocorrelation indeed shows 20-cycle oscillations with a triangular envelope, which can be understood only by taking into account the magnetic field distribution of the undulator. Therefore, one can conclude that the autocorrelation measurement provides confirmation of the 10-cycle waveform of wave packet emitted from the undulator.

### Characterization of tandem undulator radiation

**Figure 2:**
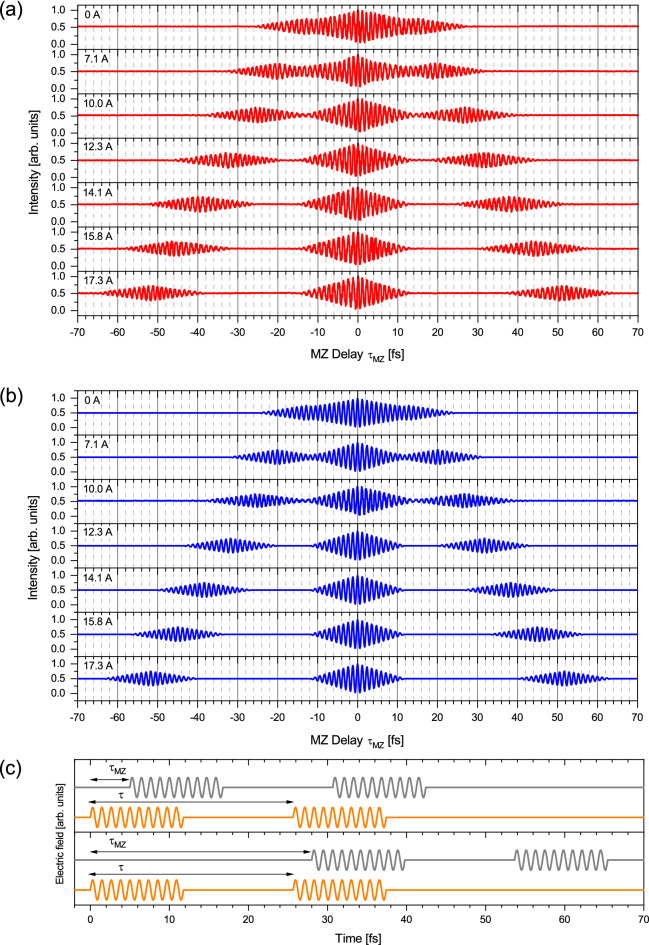
Autocorrelation measurements of spontaneous radiation from the tandem undulator at various values of the phase shifter current. The wavelength of the fundamental undulator radiation was set to 357 nm. (**a**) Measurement. (**b**) Calculation. (**c**) Waveform of the double pulsed wave packet (orange) and its delayed replica (gray) used in the calculation. As an example, the waveforms for a phase shifter current of 10.0 A is shown. The MZ delays are 5 fs and 28 fs in the top and bottom panels, respectively.

Next, we measured the autocorrelation traces for the spontaneous radiation from the tandem undulator. Figure 2a shows the interferograms obtained by setting the field strength of the phase shifter magnet to seven different values. The central wavelength of the radiation was 357 nm for both undulators. The interferograms exhibit oscillations with features which vary with the phase shifter current. The interferograms measured at a phase shifter current of 7.1 A or more show approximately 20-cycle oscillations with triangular envelopes in the range of ${\tau }_{MZ}$=−12 to  + 12 fs, as well as weaker 20-cycle oscillations lying on both sides. The side oscillation structures approach the central structure as the phase shifter current decreases, and they are unified at a phase shifter current of 0 A.

**Figure 3:**
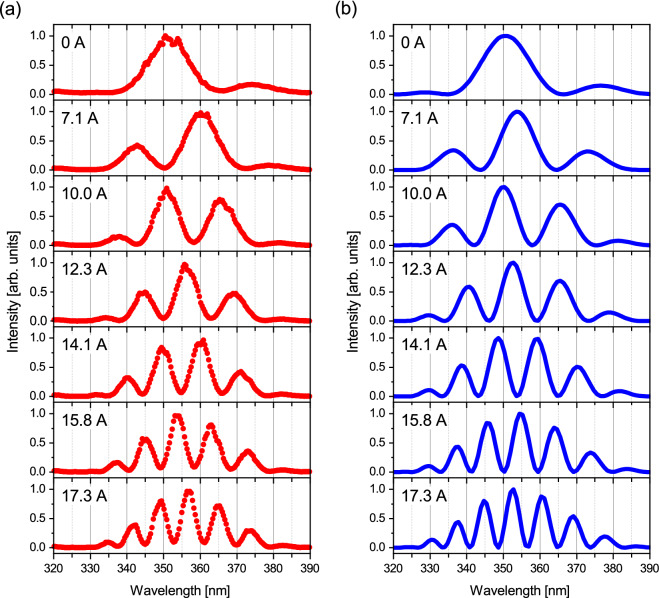
Spectra from the tandem undulator at various values of the phase shifter current. (**a**) Measured spectra. (**b**) Calculated spectra obtained by applying the Fourier transform to the double-pulsed 10-cycle sinusoidal wave packet which has the same shape used in the calculation of the autocorrelation function.

The observed interferograms can be understood as autocorrelation traces of double-pulsed wave packet. Figure 2c illustrates the two double-pulsed wave packets from the two arms in the MZ interferometer. When the relative delay ${\tau }_{MZ}$ between the two double-pulsed wave packets is shorter than the duration of each component in the double-pulsed wave packet, overlap occurs at both components of the double-pulsed wave packet, as depicted in Fig. 2c, top panel. This overlap forms the central structure observed in the interferograms. At some longer ${\tau }_{MZ}$, the first component of one of the double-pulsed wave packet overlaps the second component of the other double-pulsed wave packet (Fig. 2c, bottom panel). This leads to side structures in the interferograms, and the peak amplitude of the oscillations is half of that in the central structure. It turns out that the double-pulsed wave packet has a mutual coherence between the first and second components of the double-pulse structure. The separation between the central and side structures corresponds to the time separation between the double-pulsed components. The separation increases with a step of approximately 6 fs in the measurements presented in Fig. 3a.

The calculated autocorrelation trances for a double-pulsed 10-cycle sinusoidal wave packet at 357 nm wavelength are shown in Fig. 2b (see “Methods” for detail). The time delays between the double-pulsed components used in the calculation are determined from the temporal separations between the central and side structures in the experimental interferograms. Over the whole range of phase shifter currents, one finds excellent agreement between the experiments and calculations. This fact confirms that each of the relativistic electron emits a double-pulsed 10-cycle wave packet which well reflects the magnetic field in the tandem undulator.

### Frequency-domain interferometry

The undulator spectrum obtained in the frequency-domain reflects the waveform of the time-domain wave packet from individual relativistic electron, since they are related to each other by the Fourier transform^18^. Thus, the waveform of the wave packet can be investigated also by measuring the frequency-domain spectrum. Figure 3a shows experimental spectra of spontaneous radiation from the tandem undulator, measured under the same conditions as in the autocorrelation measurements. The spectra show interference structures which strongly depend on the phase shifter current. The number of interference fringes increases and their spacing becomes narrower with increasing phase shifter current. The observed spectral features can be interpreted in terms of the optical interference between the double-pulsed components of the wave packet. The spectral modulation reflects the interference effects for individual Fourier components contained in the undulator radiation and strongly depends on the time delay. Constructive (destructive) interference occurs when the delay length is an integer (half-integer) multiple of the wavelength^21^. As the time delay becomes longer, two neighboring wavelengths that satisfy the constructive interference condition become closer. Therefore, in Fig. 3a, as the phase shifter current increases, the spacing between fringes becomes narrower and thus the number of fringes increases.

Figure 3b shows the interference spectra calculated by assuming a double-pulsed 10-cycle sinusoidal wave packet, as in the calculation of autocorrelation functions. Applying the Fourier transform to the double-pulsed wave packet, we obtained the interference spectra in the frequency-domain. In the calculation, the time delays determined by the autocorrelation measurement were used. It is found that the experimentally observed interference structures are almost reproduced by the calculation, confirming the generation of double-pulsed wave packets from the aspect of the frequency-domain measurement. Even though reasonable agreement is obtained, there are small discrepancies in the fringe positions at phase shifter currents of 7.1 and 17.3 A. We suspect that the actual time delays probably deviated from the corresponding time-domain measurements shown in Fig. 2, due to the magnetic hysteresis of the phase shifter magnet.

### XUV Time-domain interferometry

**Figure 4:**
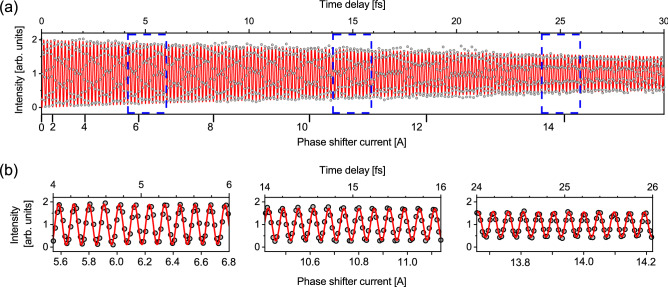
Time-domain Ramsey fringe spectrum. (**a**) The fluorescence yield in the 1 s → 5p excitation of the helium atom was measured as a function of the phase shifter current. The time delay produced by the phase shifter was calibrated using a sinusoidal curve that oscillates at 172 as period corresponding to the resonant frequency of the 1 s → 5p excitation. The time delay converted from the phase shifter current is shown on the top axis. (**b**) Enlarged plots in the three regions indicated by the blue broken lines in (**a**).

Double-pulsed structures can be expected for wave packets generated in every wavelength range covered by the tandem undulator. Here, we characterize the waveform of wave packets with XUV wavelength. Whereas autocorrelation measurements are straightforward for characterization of the waveform at UV wavelengths, application to the XUV regime is technically very difficult. Instead of a fully-optical method, we adopt a time-domain approach based on the quantum interference of electron wave packets produced in helium atoms. We measured the fluorescence yield in the 1s5p photoexcitation of helium atoms as a function of the phase shifter current, as shown in Fig. 4. This measurement is basically the same as those we have reported already^15,18^, but here we utilize the measurement to accurately determine the time delay between the pulses of the double-pulsed wave packets. The fluorescence yield spectrum shows a clear interference structure, which can be attributed to the time-domain Ramsey fringes originating from the quantum interference between the electron wave packets launched at two different times^22–24^. Because the Ramsey fringe has a time period that corresponds to the transition frequency between the ground and 1s5p states, this result demonstrates that we can measure the time delay in the 10-femtosecond regime with temporal resolution much shorter than the optical period of about 172 as.

The fringe contrast slowly decreases with increasing time delay. This can be explained by the electron beam properties, which induce a temporal spread in the time delay^16^. In order to reproduce this temporal spread, we have applied a time-damped sinusoidal curve to the delay calibration. As can be seen in the enlarged views in Fig. 4b, the time-damped sinusoidal curve fits the time-domain Ramsey fringe spectrum well. The time delay derived from the fitting analysis is shown on the top axis of Fig. 4. Here, the time delay is a relative value measured with respect to the time delay at a phase shifter current of 0 A, and corresponds to the delay produced by the phase shifter. To obtain the absolute time delay, the minimum time delay must be added.

**Figure 5:**
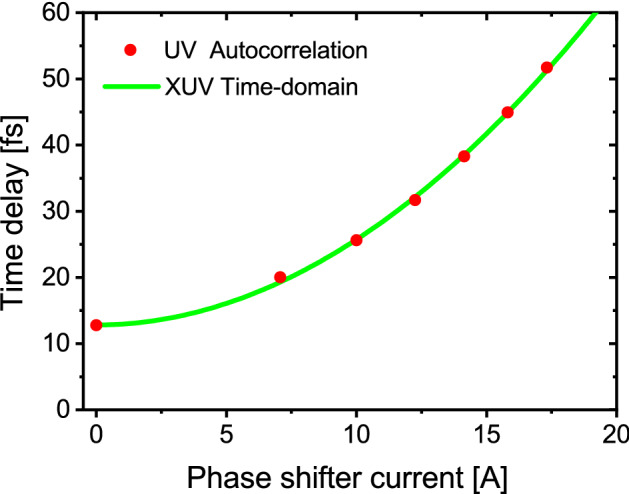
Time delay between the double-pulsed components at 357-nm wavelength obtained by the two different approaches. For comparison, the relative delay obtained in the time-domain interferometry with the XUV wave packets is normalized to the absolute delays in the UV wavelength at 0 A phase shifter current.

The time delays calibrated by the Ramsey fringe measurements are plotted in Fig. 5 together with those obtained by the autocorrelation measurement in the UV regime. Here for comparing the results from the different wavelength regimes, the relative time delay measured in the XUV wavelength was normalized to the time delay in the autocorrelation measurement at 0 A phase shifter current. The time delay varies quadratically with the phase shifter current, in accordance with the well-known behavior of the phase shifter magnet^21^. Excellent agreement is observed between the independent measurements at the two different wavelengths, providing a confirmation that the double-pulsed wave packet is emitted from each individual electron passing though the tandem undulator, and that its waveform is defined by the undulating motion of the relativistic electron.

## Conclusion

We have characterized the temporal profiles of wave packets emitted from relativistic electrons passing through a tandem undulator at a synchrotron light source. The research presented here demonstrates the ability of synchrotron light sources to control radiation waveforms at nanometer or Angstrom scales by tailoring the magnetic field distribution on the centimeter scale. We believe that making use of these unique properties of radiation provided by synchrotron light sources has the potential to open up new areas of research in photon and materials sciences. Particularly in the x-ray regime, this method could allow unprecedented control of inner-shell electron dynamics^17^, making it possible to probe specific atomic sites in a material with attosecond temporal resolution.

## Methods

### Tandem undulator and beamline

**Figure 6:**
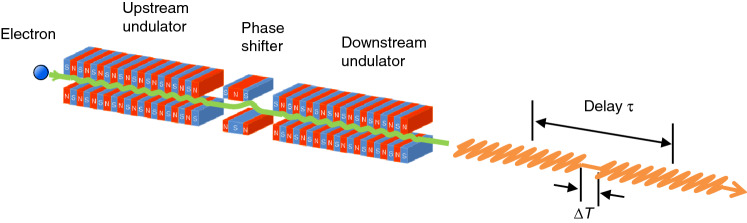
Schematic illustration of the tandem undulator. The period length and number of periods for each undulator is 88 mm and 10, respectively. A relativistic electron passing through the undulators emits a wave packet with a waveform expected to be characterized by time-separated 10-cycle oscillations. The time delay $\tau$ between the double-pulsed components of the wave packet is controlled at the attosecond level by changing the field strength of the phase shifter magnet located between the two undulators.

The experiments presented in this paper were carried out at the undulator beamline BL1U of the UVSOR III synchrotron facility, operating at an energy of 750 MeV with an emittance of 17.5 nm·rad^25^. The light source of BL1U consists of two identical APPLE-II undulators, between which a three-pole electromagnetic phase shifter is installed (Fig. 6). The period length and number of periods for each undulator were 88 mm and 10, respectively, and both undulators were set to linear polarization mode during the measurements. The electron beam current was around 20 mA, which corresponds to approximately 10^9^ electrons in each of 16 electron bunches. The double-pulsed wave packets can be expected to be randomly distributed within the overall 300 ps radiation pulse corresponding to the length of the electron bunch. The time delay $\tau$ between the double-pulsed components can be controlled by adjusting the length of electron orbit between the undulators using the phase shifter magnet. Here the time delay $\tau$ is defined by the sum of the duration of the 10-cycle oscillations and the time separation $\Delta$*T*.

We did not use a monochromator in the beamline to avoid deformation of the wave packet characterized by the double-pulsed 10-cycle oscillations. In the UV wavelength measurement, the wave packets generated by the undulators were reflected by an Al mirror, which is located at 7 m downstream from the middle point of the two undulators, and extracted to air through an Al_2_O_3_ window. In the XUV time-domain measurement, the spatially central part of the undulator radiation was cut out by a 0.4-mm-diameter pinhole and focused by a toroidal mirror. The pinhole and toroidal mirror are located at 9 and 10 m downstream from the middle point of the two undulators, respectively.

### Autocorrelation measurement

**Figure 7:**
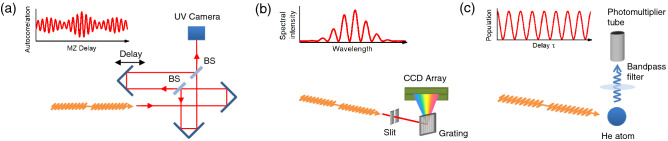
Experimental approaches used for characterizing the double-pulsed wave packet. (**a**) Autocorrelation measurement of the undulator radiation using a Mach–Zehnder interferometer in the UV regime. (**b**) Frequency-domain interferometry in the UV regime. The interference spectrum of radiation from the two undulators is recorded by a grating spectrometer. (**c**) Time-domain interferometry in the XUV regime. The fluorescence photons emitted from the 1s5p excited state of helium atom are detected by a photomultiplier tube equipped with a bandpass filter.

We measured the first order autocorrelation of undulator radiation using the MZ interferometer (Fig. 7a). The measurement was carried out in the UV wavelength owing to the ease of implementing the MZ interferometer. The wavelength of the fundamental undulator radiation was tuned to 357 nm. The MZ interferometer consists of Al mirrors (PF05-03-F01, Thorlabs), beam splitters (BSW20R, Thorlabs) and a UV camera (DMK33UP1300, The Imaging Source). The autocorrelation was measured by varying the time delay (MZ delay: τ_MZ_) of one of the two split components in the interferometer with respect to the other. The position of the delay unit was controlled by a slide stage (FS-1020PX, SIGMAKOKI) and was varied with 10 nm step, corresponding to a delay step of 0.067 fs. In the measurement, a delay range of ± 100 fs was sampled. The delay range is much longer than the temporal duration of the double-pulsed wave packet. The measured autocorrelation was normalized to the maximum amplitude at zero delay.

### Autocorrelation function of double-pulsed wave packet

The autocorrelation function for a double-pulsed *N*-cycle wave packet from the tandem undulator can be obtained for two cases. When the temporal separation Δ*T* is shorter than the duration *NT*, the autocorrelation function is expressed by

2 $$\begin{array}{c}F\left({\tau }_{MZ}\right)\propto {\int }_{-\infty }^{+\infty }{\left|E\left(t\right)+E\left(t-\tau \right)+E\left(t-{\tau }_{MZ}\right)+E\left(t-\tau -{\tau }_{MZ}\right)\right|}^{2}dt=\\ \left\{\begin{array}{c}\begin{array}{c}\begin{array}{cc}2NT& NT+\tau \le {\tau }_{MZ}\\ 2NT+\left\{NT+\left(\tau -{\tau }_{MZ}\right)\right\}\mathrm{cos}\left\{\omega \left(\tau -{\tau }_{MZ}\right)\right\}-\frac{1}{\omega }\mathrm{sin}\omega \left(\tau -{\tau }_{MZ}\right)& \tau \le {\tau }_{MZ}<NT+\tau \\ 2NT+\left\{NT-\left(\tau -{\tau }_{MZ}\right)\right\}\mathrm{cos}\left\{\omega \left(\tau -{\tau }_{MZ}\right)\right\}+\frac{1}{\omega }\mathrm{sin}\omega \left(\tau -{\tau }_{MZ}\right)& NT\le {\tau }_{MZ}<\tau \end{array}\\ \begin{array}{cc}\begin{array}{c}2NT+2\left(NT-{\tau }_{MZ}\right)\mathrm{cos}{\omega \tau }_{MZ}+\left\{NT+\left(\tau -{\tau }_{MZ}\right)\right\}\mathrm{cos}\left\{\omega \left(\tau -{\tau }_{MZ}\right)\right\}\\ +\frac{2}{\omega }\mathrm{sin}\omega {\tau }_{MZ}+\frac{1}{\omega }\mathrm{sin}\left\{\omega \left(\tau -{\tau }_{MZ}\right)\right\}\end{array}& \tau -NT\le {\tau }_{MZ}<NT\\ 2NT+2\left(NT-{\tau }_{MZ}\right)\mathrm{cos}{\omega \tau }_{MZ}+\frac{2}{\omega }\mathrm{sin}\omega {\tau }_{MZ}& 0\le {\tau }_{MZ}<\tau -NT\\ 2NT+2\left(NT-{\tau }_{MZ}\right)\mathrm{cos}{\omega \tau }_{MZ}-\frac{2}{\omega }\mathrm{sin}\omega {\tau }_{MZ}& -\left(\tau -NT\right)\le {\tau }_{MZ}<0\\ \begin{array}{c}2NT+2\left(NT-{\tau }_{MZ}\right)\mathrm{cos}{\omega \tau }_{MZ}+\left\{NT+\left(\tau -{\tau }_{MZ}\right)\right\}\mathrm{cos}\left\{\omega \left(\tau -{\tau }_{MZ}\right)\right\}\\ +\frac{2}{\omega }\mathrm{sin}\omega {\tau }_{MZ}+\frac{1}{\omega }\mathrm{sin}\left\{\omega \left(\tau -{\tau }_{MZ}\right)\right\}\end{array}& -NT\le {\tau }_{MZ}<-\left(\tau -NT\right)\\ 2NT+\left\{NT-\left(\tau +{\tau }_{MZ}\right)\right\}\mathrm{cos}\left\{\omega \left(\tau +{\tau }_{MZ}\right)\right\}+\frac{1}{\omega }\mathrm{sin}\omega \left(\tau +{\tau }_{MZ}\right)& -\tau \le {\tau }_{MZ}<-NT\\ 2NT+\left\{NT+\left(\tau +{\tau }_{MZ}\right)\right\}\mathrm{cos}\left\{\omega \left(\tau +{\tau }_{MZ}\right)\right\}-\frac{1}{\omega }\mathrm{sin}\omega \left(\tau +{\tau }_{MZ}\right)& -NT-\tau \le {\tau }_{MZ}<-\tau \\ 2NT& {\tau }_{MZ}<-NT-\tau .\end{array}\end{array}\end{array}\right.\end{array}$$

When the temporal separation is much shorter than the duration, the autocorrelation function consists of approximately 4*N*-cycle oscillations with a triangular envelope. As the temporal separation increases, the autocorrelation function splits into to the main and secondary structures (see Fig. 2). Further, when the temporal separation Δ*T* is larger than the duration *NT*, the autocorrelation function is given by

$$F\left({\tau }_{MZ}\right)\propto {\int }_{-\infty }^{+\infty }{\left|E\left(t\right)+E\left(t-\tau \right)+E\left(t-{\tau }_{MZ}\right)+E(t-\tau -{\tau }_{MZ})\right|}^{2}dt$$

3 $$=\left\{\begin{array}{c}\begin{array}{c}\begin{array}{cc}2NT& NT+\tau \le {\tau }_{MZ}\\ 2NT+\left\{NT+\left(\tau -{\tau }_{MZ}\right)\right\}\mathrm{cos}\left\{\omega \left(\tau -{\tau }_{MZ}\right)\right\}-\frac{1}{\omega }\mathrm{sin}\omega \left(\tau -{\tau }_{MZ}\right)& \tau \le {\tau }_{MZ}<NT+\tau \\ 2NT+\left\{NT-\left(\tau -{\tau }_{MZ}\right)\right\}\mathrm{cos}\left\{\omega \left(\tau -{\tau }_{MZ}\right)\right\}+\frac{1}{\omega }\mathrm{sin}\omega \left(\tau -{\tau }_{MZ}\right)& \tau -NT\le {\tau }_{MZ}<\tau \\ 2NT& NT\le {\tau }_{MZ}<\tau -NT\\ 2NT+2\left(NT-{\tau }_{MZ}\right)\mathrm{cos}\omega \tau +\frac{2}{\omega }\mathrm{sin}\omega {\tau }_{MZ}& 0\le {\tau }_{MZ}<NT\end{array}\\ \begin{array}{cc}2NT+2\left(NT+{\tau }_{MZ}\right)\mathrm{cos}\omega {\tau }_{MZ}-\frac{2}{\omega }\mathrm{sin}\omega {\tau }_{MZ}& -NT\le {\tau }_{MZ}<0\\ 2NT& NT-\tau \le {\tau }_{MZ}<-NT\\ 2NT+\left\{NT-\left(\tau +{\tau }_{MZ}\right)\right\}\mathrm{cos}\left\{\omega \left(\tau +{\tau }_{MZ}\right)\right\}+\frac{1}{\omega }\mathrm{sin}\left\{\omega \left(\tau +{\tau }_{MZ}\right)\right\}& -\tau \le {\tau }_{MZ}<NT-\tau \\ 2NT+\left\{NT+\left(\tau +{\tau }_{MZ}\right)\right\}\mathrm{cos}\left\{\omega \left(\tau +{\tau }_{MZ}\right)\right\}-\frac{1}{\omega }\mathrm{sin}\left\{\omega \left(\tau +{\tau }_{MZ}\right)\right\}& -NT-\tau \le {\tau }_{MZ}<-\tau .\\ 2NT& {\tau }_{MZ}<-NT-\tau .\end{array}\end{array}\end{array}\right.$$

The autocorrelation function consists of three parts and each of which consists of 2*N*-cylce oscillations with a triangular envelope. The temporal separation between the autocorrelation structures corresponds to the time delay τ.

### Frequency-domain interferometry

Frequency-domain interferograms were recorded using a grating spectrometer (USB2000 + , Ocean Optics) which covers the UV to near IR wavelength regime (Fig. 7b). The spectrometer consists of a 5-μm slit, a 600 lines/mm grating, collimating and focusing mirrors (not shown in Fig. 7b) and a CCD array detector and has a spectral resolution of 0.95 nm (FWHM). The wavelength of the fundamental undulator radiation was set to 357 nm. Similar to the autocorrelation measurement, the undulator radiation was extracted to air through the Al_2_O_3_ window and was introduced to the spectrometer. The mutual coherence between the double-pulsed components in the wave packet induces an interference structure in the spectrum. Assuming that the two undulators are identical, the spectrum of tandem undulator is given by^20,21^

4 $$I\left(\lambda \right)={I}_{0}\left(\lambda \right)\left\{1+{f}_{mod}\mathrm{cos}\left(2\pi \frac{c\tau }{\lambda }\right)\right\}$$

here *I*_0_($\lambda$) is the spectrum of a single undulator, *f*_mod_ is a contrast of interference fringe and *c* is the speed of light.

### XUV time-domain interferometry

We carried out a time-domain interferometric measurement in the XUV wavelength region. For the measurements, we set the wavelength of fundamental undulator radiation to 51 nm, and arbitrarily tuned the time delay $\tau$ in the 10-femtosecond regime. In the time-domain interferometry, we measured the fluorescence intensity emitted from helium atoms irradiated by the undulator radiation, as shown in Fig. 7c. The sequential interaction between the atomic target and the double pulse induces quantum interference in electron wave packets^22^. Since the wave packet consists of a superposition of eigenstates, the quantum interference results in a sinusoidal modulation of eigenstate population dependent on the time delay

5 $${n}_{k}\propto 1+\mathrm{cos}\left({\omega }_{k}\tau +{\phi }_{k}\right)$$

where *ω*_k_ is the resonant angular frequency of the eigenstate and ϕ_k_ is the phase of the eigenfunction. Although the amplitude and phase of each frequency component of the optical pulse are imprinted on the electron wave packet^24^, characterization of the optical pulse only by the population measurement is a rather difficult task due to the requirement on precise knowledge of several parameters such as excitation cross sections and detection efficiencies of the eigenstates included in the wave packet. Thus, we only measured the time delay between the double-pulsed components.

The focused undulator radiation interacts with an effusive helium gas beam. A 75-nm thickness Al filter was inserted in front of the experimental chamber to eliminate the influence of UV stray light. The fluorescence photons of 361-nm-wavelength emitted from the 1s5p state were selectively detected by using a photomultiplier tube (R6249P, Hamamatsu) equipped with a bandpass filter (HQBP360-UV, Asahi Spectra). We observed the modulation of the 1s5p excited state population, the so called “time-domain Ramsey fringes” in the photoexcitation of helium atoms^15^. Since the Ramsey fringes have a time period corresponding to the transition frequency, the time delay can be determined with sub-cycle i.e., attosecond accuracy, which is much better than that achieved in the UV regime, according to the reference value in the high-precision atomic spectroscopy^26^. For calibration, the Ramsey fringe spectrum was fitted by a time-damped sinusoidal curve to reproduce the decrease of the fringe contrast with increasing time delay. In the fitting, we assumed that the delay is proportional to *I*^m^, where *I* is the phase shifter current, and obtained *m* = 1.99, which agrees well with the theoretical value of *m* = 2^21^.

## Data Availability

The data that support the findings of this study are available from the corresponding author upon reasonable request.

## References

[1] Onuki H, Elleaume P (2003). Undulators, Wigglers and Their Applications.

[2] Sawhney KJS (1997). A novel undulator-based PGM beamline for circularly polarised synchrotron radiation at BESSY II. Nucl. Inst. Methods. A.

[3] Muro T (2005). Circular dichroism measurement of soft X-ray absorption using helicity modulation of helical undulator radiation. J. Electron Spectrosc. Relat. Phenom..

[4] Amemiya K (2013). Fast polarization switching in the soft X-ray region at PF BL-16A. J. Phys. Conf. Ser..

[5] Follath R (2013). The energy materials in-situ laboratory Berlin (EMIL) at BESSY II. J. Phys. Conf. Ser..

[6] Lee T-L, Duncan DA (2018). A two-color beamline for electron spectroscopies at Diamond Light Source. Synchrot. Radiat. News.

[7] Kim KJ (1984). A synchrotron radiation source with arbitrarily adjustable elliptical polarization. Nucl. Inst. Methods..

[8] Bahrdt J (1992). Circularly polarized synchrotron radiation from the crossed undulator at BESSY. Rev. Sci. Instrum..

[9] Matsuda I, Yamamoto S, Miyawaki J, Abukawa T, Tanaka T (2019). Segmented undulator for extensive polarization controls in ≤1 nm-rad emittance rings. J. Surface Sci. Nanotechnol..

[10] Miyawaki J (2021). Fast and versatile polarization control of X-ray by segmented cross undulator at SPring-8. AAPPS Bull..

[11] Bahrdt J (2013). First observation of photons carrying orbital angular momentum in undulator radiation. Phys. Rev. Lett..

[12] Katoh M (2017). Helical phase structure of radiation from an electron in circular motion. Sci. Rep..

[13] Kaneyasu T (2017). Observation of an optical vortex beam from a helical undulator in the XUV region. J. Synchrotron Radiation.

[14] Matsuba S (2018). Generation of vector beam with tandem helical undulators. Appl. Phys. Lett..

[15] Hikosaka Y, Kaneyasu T, Fujimoto M, Iwayama H, Katoh M (2019). Coherent control in the extreme ultraviolet and attosecond regime by synchrotron radiation. Nat. Commun..

[16] Kaneyasu T, Hikosaka Y, Fujimoto M, Iwayama H, Katoh M (2019). Controlling the orbital alignment in atoms using cross-circularly polarized extreme ultraviolet wave packets. Phys. Rev. Lett..

[17] Kaneyasu T, Hikosaka Y, Fujimoto M, Iwayama H, Katoh M (2021). Electron wave packet interference in atomic inner-shell excitation. Phys. Rev. Lett..

[18] Hikosaka Y, Kaneyasu T, Fujimoto M, Iwayama H, Katoh M (2021). Reply to ‘Comment on “Coherent control in the extreme ultraviolet and attosecond regime by synchrotron radiation”’. Nat. Commun..

[19] Loudon R (1983). The quantum theory of light.

[20] Kaneyasu T, Hikosaka Y, Fujimoto M, Iwayama H, Katoh M (2020). Polarization control in a crossed undulator without a monochromator. New J. Phys..

[21] Elleaume P (1983). Optical Klystrons. J. Phys. Colloques.

[22] Bouchene MA (1998). Temporal coherent control induced by wave packet interferences in one and two photon atomic transitions. Eur. Phys. J. D.

[23] Rulliére C (2005). Femtosecond Laser Pulses.

[24] Ohmori K (2009). Wave-packet and coherent control dynamics. Annu. Rev. Phys. Chem..

[25] Adachi M (2013). Design and Construction of UVSOR-III. J. Phys. Conf. Series.

[26] Kandula DZ (2011). XUV frequency-comb metrology on the ground state of helium. Phys. Rev. A.

